# The Reciprocal Relationship Between Gratitude and Life Satisfaction: Evidence From Two Longitudinal Field Studies

**DOI:** 10.3389/fpsyg.2019.02480

**Published:** 2019-11-08

**Authors:** Wenceslao Unanue, Marcos Esteban Gomez Mella, Diego Alejandro Cortez, Diego Bravo, Claudio Araya-Véliz, Jesús Unanue, Anja Van Den Broeck

**Affiliations:** ^1^Escuela de Negocios, Universidad Adolfo Ibáñez, Santiago, Chile; ^2^Escuela de Psicología, Universidad Adolfo Ibáñez, Santiago, Chile; ^3^Facultad de Educación y Ciencias Sociales, Universidad Andres Bello, Santiago, Chile; ^4^KU Leuven, Leuven, Belgium; ^5^Optentia Research Programme, North-West University, Vanderbijlpark, South Africa

**Keywords:** gratitude, life satisfaction, subjective well-being, positive psychology, longitudinal analysis, prospective design, adults, Chile

## Abstract

Gratitude and life satisfaction are associated with several indicators of a good life (e.g., health, pro-social behavior, and relationships). However, how gratitude and life satisfaction relate to each other over time has remained unknown until now. Although a substantial body of research has tested the link from gratitude to life satisfaction, the reverse association remains unexplored. In addition, recent cross-cultural research has questioned the link between gratitude and subjective well-being in non-Western countries, suggesting that the benefits of gratitude may only prevail in Western societies. However, previous cross-cultural studies have only compared western (e.g., American) and eastern (e.g., Asian) cultures, but this simple contrast does not adequately capture the diversity in the world. To guide further theory and practice, we therefore extended previous cross-sectional and experimental studies, by testing the bi-directional longitudinal link between gratitude and life satisfaction in a Latin American context, aiming to establish temporal precedence. We assessed two adult samples from Chile, using three-wave cross-lagged panel designs with 1 month (Study 1, *N* = 725) and 3 months (Study 2, *N* = 1,841) between waves. Both studies show, for the first time, that gratitude and life satisfaction mutually predict each other over time. The reciprocal relationships suggest the existence of a virtuous circle of human well-being: higher levels of gratitude increase life satisfaction, which in turn increases gratitude, leading to a positive spiral. Key theoretical and practical implications for the dynamics of human flourishing and field of positive psychology are discussed.

Thanks to life, which has given me so much

It gave me two stars, which when I open them,

Perfectly distinguish black from white

And in the tall sky its starry backdrop,

And within the multitudes the one that I love.

Thanks to life

Violeta Parra, Chilean poet

Life satisfaction and gratitude are important for living a good life. The benefits of both constructs have been extensively documented. They include, for instance, better mental and physical health, more pro-social behavior, high-quality relationships, and more meaningful lives (Wood et al., 2010; Diener and Tay, 2017). Life satisfaction (Diener, 1984) is a key predictor of well-being (Helliwell et al., 2013) and a fundamental construct for advising on public policies (Diener et al., 2009): the OECD, e.g., has used life satisfaction to assess the progress of the nations through the Better Life Index (OECD, n.d.). Gratitude, a tendency to appreciate the good and positive, is an equally essential nutrient for people flourishing (Wood et al., 2010).

Research has extensively shown a positive link between gratitude and life satisfaction (Froh et al., 2009; Wood et al., 2010; Alkozei et al., 2018). However, how both constructs relate to each other over time has remained unknown until now. Previous studies have only explored the link from gratitude to life satisfaction, whereas the reverse association has not been tested yet. Drawing on Watkins (2004) seminal article, we theorized a reciprocal relationship between both constructs and thus a “circle of virtue”.

As gratitude and life satisfaction likely unfold over time, we need to do more to disentangle the ongoing, naturally occurring, reciprocal relations between pre-existing (rather than momentarily primed) gratitude and life satisfaction. Appropriate and well-suited longitudinal designs—still scarce in the field—are needed in order to complement the existing evidence and test whether both constructs are reciprocally related (Wood et al., 2008). This paper presents two such studies, among Chilean adults, that could contribute in this area.

Studying the directionality between gratitude and life satisfaction is important, both from a theoretical and practical point of view. From a theoretical perspective, our studies make four main contributions. First, longitudinal field research is necessary for clarifying the direction of the link between gratitude and life satisfaction, in order to identify whether there is a temporal precedence between the constructs or whether the link is only due to a shared variance with other variables. Second, clarifying the prospective direction of the link between gratitude and life satisfaction allows their conceptualizations and implications to be enriched. If our reciprocal hypothesis is supported, gratitude would be not only an antecedent of life satisfaction but also a consequence of it and vice versa. These findings would show the complexity, multi-directionality, and interdependence between both constructs. Third, the potential influence of gratitude on subjective well-being (SWB; Diener, 1984) has not yet been fully confirmed in the non-Western world. Indeed, recent cross-cultural research has suggested that benefits of gratitude may only reach Western societies (Boehm et al., 2011; Layous et al., 2013; Shin et al., in press). However, previous studies have only compared Asian and American cultures. Therefore, we think it is important to extend gratitude research by including additional non-Western countries like Chile, which allows us to go beyond the traditional Western-Eastern dichotomy (Vignoles et al., 2016). Fourth, while the great majority of previous studies have explored students and young populations (Davis et al., 2016), we assessed working adults.

From a practical point of view, if the reciprocal relationship is supported, it would open the possibility for a virtuous or a vicious circle in health and well-being interventions. On the one hand, higher gratitude would lead to higher life satisfaction, which in turn would increase gratitude, leading to a positive spiral in human flourishing. On the other hand, the lack of either gratitude or life satisfaction may lead to a negative process in human wellness. Policy makers and health practitioners could benefit from these findings. By teaching people the importance of gratitude and life satisfaction—and how to foster each of them, practitioners from different settings (clinical, educational, organizational, etc.) may not only help people to protect their mental health but also show them how to move toward a virtuous circle of flourishing and well-being.

Accordingly, we conducted two longitudinal studies to examine the prospective link from gratitude to life satisfaction as well as the reverse link from life satisfaction to gratitude. Before presenting the results, we first describe gratitude and life satisfaction and argue for their reciprocal relationship.

## Gratitude and Life Satisfaction

Gratitude has been conceptualized from different perspectives (McCullough et al., 2002). The most comprehensive approach—and the one we used in this paper–defines gratitude as a life orientation (Wood et al., 2010). From this perspective, people may feel grateful because they are alive, because they are able to walk in a beautiful park, or just from the appreciations of their abilities (Wood et al., 2010). Research has found that higher gratitude is associated with a better life, indexed as higher positive affect, self-esteem, positive emotions, optimism, autonomy, environmental mastery, relationships, personal growth, meaning in life, and self-acceptance. Gratitude has also been associated with lower ill-being in terms of negative affect, depression, anxiety, phobia, bulimia, addictions, negative emotions, dysfunctions, anger, and hostility. For a review and a meta-analysis, see Davis et al. (2016) and Wood et al. (2010).

Subjective well-being (SWB; Diener, 1984) refers to “people’s sense of wellness in their lives, in both thoughts and feelings” (Diener and Tay, 2017, p. 90). Life satisfaction is the cognitive component of SWB (Diener et al., 1985) and reflects the global evaluation that people make about their satisfaction with their own lives in several domains such as work, marriage, and health (Diener et al., 2017). Life satisfaction is associated with a host of positive outcomes, indexed in terms of better mental and physical health, healthier weight and eating behaviors, more exercise, longer life expectancy, higher levels of career satisfaction, lower turnover intentions, and higher organizational commitment. It has also been associated with lower ill-being, indexed as lower addictions and unhealthy habits (e.g., tobacco, drugs, and alcohol use), lower mortality rates, and lower levels of anxiety and depression. The benefits of life satisfaction also reach the whole of society. Higher life satisfaction predicts altruism (e.g., donating, helping, and volunteering) as well as lower homicide, suicide, and illness rates. For a review, see Diener et al. (2017) and Diener and Tay (2017).

### Research Studying the Link Between Gratitude and Life Satisfaction: The Need for Longitudinal Studies

Cross-sectional studies have given strong support for the relationships between gratitude and life satisfaction. However, cross-sectional designs are not able to disentangle either the origins or the direction of this relationship. Experimental evidence has found support for the hypothesized causal link from gratitude to life satisfaction. Priming or experimentally inducing gratitude leads participants to feel better about their lives as a whole and to experience more life satisfaction (Emmons and McCullough, 2003; Rash et al., 2011). Writing letters of gratitude over a 3-week period also increases participants’ happiness and life satisfaction and decreases depressive symptoms (Toepfer et al., 2012). Experimental studies are the strongest evidence for causality between gratitude and life satisfaction. However, previous research has focused only on the effect of gratitude on life satisfaction, yet no experimental study to date has tested a reverse link. Longitudinal research may help to fill this gap.

Although longitudinal studies have examined several aspects of the prospective relations of gratitude, such as social support, low stress, or post-traumatic growth (Wood et al., 2008; Zhou and Wu, 2016), according to our knowledge, only one field study has explored the link between gratitude and life satisfaction over time, using an appropriate longitudinal design. Specifically, Jans-Beken et al. (2018) found a prospective positive association from gratitude to SWB, using a four-wave design among Dutch adults. However, only a global measure of SWB was included and life satisfaction was not isolated. Importantly, the reverse link from life satisfaction to gratitude was neither hypothesized nor tested. Longitudinal research using questionnaires would help to extend previous cross-sectional and experimental evidence and shed light on the hypothesized prospective link between gratitude and life satisfaction. Conducting this kind of study is the main aim of our paper.

## The Reciprocal Relation Between Gratitude and Life Satisfaction

We contend that gratitude and life satisfaction may be reciprocally related. The idea was first developed by Watkins (2004), who proposed several psychological mechanisms to understand the so-called “circle of virtue.” Below, we will summarize some of his main ideas.

### From Gratitude to Life Satisfaction

Gratitude is a life orientation towards noticing and appreciating the positive in life: it “serves as an indicator of aspects of life for which to be appreciative” (Wood et al., 2010, p. 3). This is a dispositional tendency. Thus, people high in trait gratitude experience all the gratitude facets frequently and strongly (McCullough et al., 2002), which may lead to positive cognitive evaluations of our existence (e.g., higher life satisfaction assessments). Watkins (2004) offered several suggestions about which psychological mechanisms are involved in the prospective link from gratitude to life satisfaction.

First, when people perceive a benefit/favor as a “gift” (i.e., “a favor that has been given to one for one’s benefit,” Watkins, 2004, p. 175), they are more likely to enjoy the benefit. This perception may be a form of cognitive amplification, which in turn fosters SWB. People higher in trait gratitude are more likely to perceive benefits as gifts, which could lead gratitude to increase life satisfaction through this cognitive amplification process. In other words, “gratitude should increase our enjoyment of a blessing” (Watkins, 2004, p. 176). This theorization is consistent with the broaden-and-build theory (BBT; Fredrickson, 2013). BBT suggests that gratitude, as a life orientation, may consistently increase our positive emotions, which in turn broadens our array of thoughts, increasing life satisfaction: when people feel grateful for a situation—especially when the situation is seen as a gift—they are more likely to feel positive emotions, and this in turn protects them from a variety of mental disorders and increases their life satisfaction and happiness (Lyubomirsky et al., 2005). This process then produces an upward spiral in human wellness (Fredrickson, 2013).

Second, gratitude may protect us against the law of habituation. Research has shown that people tend to adapt to their current levels of circumstances, and “over time, we tend to get used to our current level of satisfaction” (Watkins, 2004, p. 176). Unfortunately, adaptation to satisfaction may prevent people from being happy from ongoing circumstances. Certain activities may help to avoid being a slave to the law of habituation. Indeed, “by constantly being aware of how fortunate one’s condition is” (e.g., through gratitude), people may protect themselves from the problem of habituation (Frijda, 1988, p. 354). In other words, the “practice of gratitude should accomplish, consistently reminding one of how good life really is” (Watkins, 2004, p. 177).

Third, gratitude may direct attention away from upward social comparisons. Social comparisons lead to feelings of deprivation. Indeed, upward social comparisons and envy is associated with lower positive affect and higher unpleasant feelings. However, as shown by McCullough et al. (2002), the practice of gratitude (e.g., focusing on our blessings), “directs attention away from making comparisons with others who have more” (Watkins, 2004, p. 177). In other words, changing our attention from the things we do not have to an appreciation of thing we do have may protect humans from the dangers of social comparisons (Watkins, 2004).

Fourth, the practice of gratitude is an effective coping mechanism. Wood et al. (2007) showed that gratitude relates to three broad categories of coping (Wood et al., 2010): People who are more grateful tend to use more social support, to actively solve their problems, and to avoid denying the existence of the problems. These coping strategies may help individuals to better face and solve various life problems, thus increasing their life satisfaction. To support this, research has shown that grateful people are better able to appreciate difficult situations, promoting better coping strategies with stressful circumstances, which is associated with long-term SWB (Watkins, 2004). In other words, “gratitude may give one a helpful perspective on life that assists in mood repair following a stressful event” (p. 179).

Fifth, gratitude allows the accessibility and recollection of pleasant life events. Seidlitz and Diener (1993) state that a key aspect of happiness is the accessibility of positive memories. Following this argument, Watkins (2004) argues that gratitude “should enhance the retrievability of positive experiences by increasing elaboration of positive information” (p. 181). Further, the increased availability of positive life events should lead to more positive judgments of people’s lives and thus to higher life satisfaction.

Sixth, gratitude may increase life satisfaction by enhancing a person’s social benefits. Indeed, whereas research has shown that gratitude is significantly associated with better social relationships (Wood et al., 2010), social relationships are strongly associated with higher life satisfaction (Unanue et al., 2014). Further, gratitude may increase life satisfaction through the mediational role played by social contacts and the satisfaction of the need for relatedness (Watkins, 2004). Seven, gratitude might increase life satisfaction through the prevention of depressive episodes. Indeed, research has shown that depression has a strong inverse association with gratitude. Because of that, it has been argued that “the lack of gratitude may be a vulnerability factor for depression” (p. 183) and thus of lower life satisfaction and SWB.

### From Life Satisfaction to Gratitude

Previous arguments provide a strong argument for the link from gratitude to life satisfaction. However, it is also possible to theorize that life satisfaction may also predict gratitude over time.

Gratitude—as a life orientation—represents satisfaction in several aspects of life such as social support, work, and family (Wood et al., 2008). Thus, when satisfaction with life increases, a causal effect is expected such that people’s gratitude increases accordingly. In other words, people may feel a strong sense of gratitude when experiencing high levels of life satisfaction (e.g., their lives are fantastic). In addition, according to Watkins (2004), research suggests that people who are satisfied with their lives develop three types of perceptions when they are the recipient of the gift, which may increase gratitude. First, people who are satisfied with their lives, are more likely to value a gift, and are therefore more likely to experience gratitude. Second, when the receiver appreciates the goodness of the giver, grateful feelings increase. Third, the receiver is more likely to feel grateful if he or she thinks that the gift is gratuitous and went beyond the receiver’s social expectations. Happier people are more likely to have the previous three perceptions, which in turn lead them to feel more grateful. Research strongly supports these claims. For example, people experiencing greater life satisfaction or positive affect tend to evaluate things more positively, which increases the probability of a grateful response. In other words, people are more likely to recognize the goodness of benefits if they believe life is good, thus promoting grateful responses.

Overall, whether gratitude causes life satisfaction, and/or life satisfaction causes gratitude, is still an open question. Following Watkins (2004) seminal article, we propose that the answer to both questions is yes. In other words, we expect that gratitude and life satisfaction operate in a “cycle of virtue” (p. 185). Based on this theorizing, we thus expect a bi-directional temporal association between gratitude and life satisfaction, and hypothesize:

(H1) Gratitude prospectively predicts future life satisfaction.

(H2) Life satisfaction prospectively predicts future gratitude.

## The Role of Culture: Extending Research in Non-Western Countries

Despite the increasing evidence in favor of a positive link between gratitude and SWB in the Western world, cross-cultural research has questioned the potential influence of gratitude in non-Western countries (Boehm et al., 2011; Layous et al., 2013; Shin et al., in press). For example, while some studies in China (Sun and Kong, 2013; Kong et al., 2015, 2017) and Philippines (Datu, 2014; Datu and Mateo, 2015; Valdez et al., 2017) have shown a positive link between gratitude and SWB, recent research in South Korea, Taiwan, and India found non-significant results.

Boehm et al. (2011) explored the effect of a gratitude intervention on life satisfaction among Anglo-American and Asian American participants. Individuals from both cultures reported higher life satisfaction after the intervention (compared with the control group), but Asian American participants benefitted significantly less. Similarly, Layous et al. (2013) studied the effect of a gratitude intervention on SWB (life satisfaction and positive emotions) among North American and South Korean participants. Results showed that SWB increased in both cultures (compared with the control group), but the increase was significantly lower for the South Korean participants. Shin et al. (in press) randomly assigned participants from India, Taiwan, and the US to a gratitude experimental condition or to a neutral condition activity. It was found that only the US participants who expressed gratitude reported a greater state of gratitude relative to the controls, which led the authors to suggest that gratitude interventions do not “elicit felt gratitude in collectivist cultures,” providing “new insights into why expressing gratitude may be a less effective happiness-promoting activity in collectivist cultures” (p. 2).

Previous findings have led scholars to argue that maybe “Eastern, collectivist cultures do not benefit as much from practicing gratitude compared to Western, individualist cultures” (Shin et al., in press, p. 2). However, existing studies exploring the role of culture in the link between gratitude and SWB and have only compared Western (e.g., US) and Eastern (e.g., Asian) cultures, which is in line with the standard tradition in cross-cultural psychology, which “has relied excessively on contrasts between North American and East Asian samples” (Vignoles et al., 2016, p. 967). Nonetheless, a simple contrast between Eastern and Western countries does not adequately capture the diversity in different regions of the world (Vignoles et al., 2016).

Markus and Kitayama (1991) proposed that cultures could be classified under two opposite dimensions: independent and interdependent. The authors stated that the *independent* view of the self is found in Western countries and the *interdependent* view of the self is found in non-Western societies. However, according to Vignoles et al. (2016), “this perspective has arguably contributed to the prevalence of a rather black-and-white view of cultural diversity” (p. 969), leading academics to legitimize a misleading tendency to dichotomize cultures in terms of binary oppositions between “Western” (e.g., US) versus “non-Western” (e.g., Asia) cultures. Further, this black-and-white view between US and Asia has marginalized other non-Western regions of the world such us Latin America, Sub-Saharan Africa, the Middle East, and Eastern Europe. Research on gratitude has made the same mistake. To fill this void, we extend cross-cultural research on gratitude beyond the East-West dichotomy by studying Chile.

Cultural diversity may be assessed through national socioeconomic development, religious heritage, and individualism (Vignoles et al., 2016). Based on these criteria, Latin America, and in particular Chile, is different from American and Asian countries studied thus far, and studying this particular context thus adds important value to the diversity of the cross-cultural research on gratitude. First, according the World Bank, Latin America is considered an upper-middle-income region, whereas North America is a high-income economy and most Asian nations are low-income ones (The World Bank, n.d.). Second, according to the World Economic Forum, Chile and Latin America have a Catholic heritage, whereas most of the population in the US is atheist/agnostic, and a large majority of people from Asia are either Buddhist, Hindu, or atheist/agnostic (Jacobs, 2019). Third, and finally, Chile is an interesting country in terms of the dimension of individualism-collectivism. Research has assumed that people from Western countries have an individualistic view of the self, while people from non-Western countries have a more collectivistic view. Following this tradition, Chile has been traditionally considered a collectivistic culture (Hofstede, 1983; Arnulf and Silje, 2009). However, during the last few decades, Chile has gone through a deep social and economic transition with enormous cultural and societal changes. Indeed, recent studies have shown that Chile has moved fast toward a more individualistic culture (Arnulf and Silje, 2009; Benavides and Hur, 2019).

Individualism is a key issue, and researchers have tried to explain why the benefits of gratitude seem only to have reached Western societies (Boehm et al., 2011; Shin et al., in press). Research has shown that “individualist cultures base their life satisfaction more on intrapersonal than interpersonal factors whereas those from collectivist cultures do the reverse” (Boehm et al., 2011, p. 2). In other words, goals and norms in individualistic cultures are more supportive of self-expression, self-improvement, and the pursuit of happiness rather than goals and norms in collectivistic cultures (Boehm et al., 2011). If individualism is key, we may expect a positive link between gratitude and SWB in Chile, which is an unexplored non-Western cultural context.

## Contributions of the Present Research

We followed Watkins (2004), in terms that “The test of all happiness is gratitude” (Watkins, 2004, p. 167). Further, he states that the relation between gratitude and happiness, and more specifically, life satisfaction, should not be taken lightly and deserves to be extensively studied. Our paper aims to tap into this issue and study the link between gratitude and life satisfaction from a longitudinal perspective.

The current manuscript contributes to the scientific literature in the following ways. First, there is a lack of well-suited longitudinal field research on the association between gratitude and life satisfaction (Alkozei et al., 2018). Indeed, according to our knowledge, to date, no study has explored the reciprocal link between these constructs using questionnaire research. In response, we conducted two field studies, using cross-lagged panel models (CLPMs) which help in testing prospective (i.e., temporal) directions between gratitude and life satisfaction over time (Selig and Little, 2012). Although prospective designs do not test causality directly, prospective significance between variables is a key requirement for causality. CLPM allows “looking at autoregressive effects (linking a variable at earlier time points to itself at later time points) and cross-lagged effects (linking two different variables across time)” (Joshanloo, 2019, p. 183).

Second, we expand on the scarce amount of research conducted in the non-Western world (mainly in Asia), by assessing a country from a Latin-American context. By including Chile, we extended previous research beyond the traditional Western-Eastern paradox (Vignoles et al., 2016). Third, the great majority of previous studies on the link between gratitude and life satisfaction have focused on students and young populations going through similar life transitions (Davis et al., 2016). We aim to further our understanding of the relationship between gratitude and life satisfaction, by exploring two large samples of Chilean working adults, living at different stages of their lifespan. Finally, our research also has practical implications. By complementing previous experimental and cross-sectional studies, we expect to test the potential of both gratitude and life satisfaction for interventions aiming to protect people’s mental health, improve the quality of human life, and provide guidance on public policies.

## Study 1

### Method

#### Participants and Procedure

Study 1 was conducted in accordance with the American Psychological Association guidelines and followed University Ethics and Research Governance procedures to avoid coercion (e.g., participation was voluntary). Participants were informed about the goal of the study in overall terms. They were also asked about their intention to participate in future research, as the poll would be part of a longitudinal study. Informed consent was obtained from all participants.

Following recent leading research, which advocates the advantages of using online designs (Porter et al., 2019), we collected full panel data in a three-wave cross-lagged longitudinal design with 1 month between waves, among a wide sample of Chilean working adults. A university in Santiago provided the email addresses of alumni^1^. Participants were sent an email with an explanation of the research and a web link to the survey. In each wave, participants were advised that the survey remained opened for only 1 week, and they received a polite reminder every working day. All participants who decided not to participate in or finish the study were given the option to either unsubscribe from the mailing list or leave the survey at their convenience, without any penalty. For the rest of the participants, all questions were compulsory, so we did not have missing data within each wave.

Seven hundred and twenty-five participants (52.1% male) between the ages of 21 and 72 years (mean age = 38.30; SD = 10.01) completed the T1 measures. At T2, 275 participants (52.7% male) between the ages of 21 and 72 years (mean age = 39.62; SD = 10.23) completed the T2 measures (37.93% of Wave 1). At T3, 252 participants (55.2% male) between the ages of 21 and 72 years (mean age = 40.35; SD = 10.15) completed the T3 measures (34.76% of Wave 1). In total, 161 respondents (54.7% male) between the ages of 21 and 72 (mean age = 40.65; SD = 10.50) answered the three waves (22.21% of Wave 1). Those who completed only T1 (*N* = 564) did not differ significantly in gender {[*χ*^2^(1)] = 0.53, *p* = 0.468}, gratitude [t(275.67) = −1.86, *p* = 0.064], or life satisfaction [t(723) = −1.02, *p* = 0.307] from those who participated in the three waves (*N* = 161). Participants only differed in age [t(723) = −3.40, *p* < 0.01]. Therefore, our analysis suggests that younger participants were especially likely to drop out of the study. Little’s MCAR test (Little, 1988) showed that missing data were completely at random {[*χ*^2^(141)] = 115.24, *p* = 0.945}. Following the recommendations of Newman (2014), we employed a full information maximum likelihood estimation (FIML^2^), which allowed us to include all 725 participants in our structural analyses, irrespective of the pattern of missing data (Muthén et al., 1987).

We conducted a sensitivity power analysis using G*Power 3.1 (Faul et al., 2009) to estimate the statistical power for our cross-lagged structural equation modeling (SEM) model. Adopting the conventional criterion of 0.80 power, considering 124 parameters, and including only participants who completed the three waves, which is a conservative criterion, our study was sufficiently powered to detect a predictor with a population effect size of *f*^2^ = 0.051, representing a small effect (Cohen, 1992). Our sample size was thus considered sufficient. The distributions were adequate for all constructs (George and Mallery, 2010). Skew values were appropriate for gratitude (T1: −0.79; T2: −0.94; and T3: −0.97) and life satisfaction (T1: −0.81; T2: −0.82; and T3: −0.92). Kurtosis values were also appropriate for gratitude (T1: 0.23; T2: 1.52; and T3: 0.74) and life satisfaction (T1: 0.58; T2: 0.94; and T3: 0.82).

#### Measures

We translated highly validated scales for gratitude and life satisfaction into Spanish, and equivalence of meaning with the original version was checked using standard back-translation procedures (Brislin, 1970)^3^.

#### Gratitude

We used the gratitude questionnaire developed by McCullough et al. (2002), which includes six items (e.g., “If I had to list everything that I felt grateful for, it would be a very long list”). Respondents rated the items from 1 (completely disagree) to 7 (completely agree). Cronbach’s alphas were good at T1 (0.76), T2 (0.74), and T3 (0.78). We built a latent variable using all the scale items.

#### Life Satisfaction

We used the Satisfaction with Life Scale (Diener et al., 1985), which includes five items (e.g., “In most ways my life is close to my ideal”). Respondents rated the items from 1 (strongly disagree) to 6 (strongly agree). Cronbach’s alphas were good at T1 (0.89), T2 (0.88), and T3 (0.88). We built a latent variable using all the scale items.

#### Demographics

We used gender (male = 1) and age (in years) as control variables.

### Results

Descriptive statistics and intercorrelations for all Study 1 variables are shown in Table 1. We used MPlus 7.1 (Muthén and Muthén, 2012) to estimate the relations among our constructs. We used SEM to test our hypotheses. We used latent variables to reduce the biasing effects of measurement error (Finkel, 1995). According to standard statistical criteria (Hu and Bentler, 1999; Kline, 2005), we evaluated the model fit by using the root mean square error of approximation (RMSEA) and comparative fit index (CFI). Values of RMSEA <0.06 (or < 0.08) and CFI > 0.95 (or > 0.90) were considered to be evidence of a good (or acceptable) fit.

**Table 1:** Descriptives and inter-correlations for all Study 1 and Study 2 variables.

*Study 1*	*M*	*D*	*1*	*2*	*3*	*4*	*5*	*6*	*7*	*8*
1. Gender	1.48	0.50								
2. Age	38.3	10.01	−0.20^**^							
3. Life satisfaction T1	4.57	0.97	0.01	0.03						
4. Life satisfaction T2	4.61	0.89	0.04	0.02	0.73^**^					
5. Life satisfaction T3	4.65	0.90	0.07	−0.01	0.72^**^	0.77^**^				
6. Gratitude T1	5.92	0.91	0.18^**^	0.03	0.50^**^	0.40^**^	0.46^**^			
7. Gratitude T2	5.95	0.87	0.19^**^	0.05	0.44^**^	0.51^**^	0.47^**^	0.72^**^		
8. Gratitude T3	5.95	0.94	0.18^**^	−0.01	0.49^**^	0.52^**^	0.60^**^	0.71^**^	0.69^**^	
***Study 2***	***M***	***D***	***1***	***2***	***3***	***4***	***5***	***6***	***7***	***8***
1. Gender	1.45	0.50								
2. Age	36.94	8.59	−0.11^**^							
3. Life satisfaction T1	4.42	1.00	0.16^**^	0.06^*^						
4. Life satisfaction T2	4.51	0.98	0.16^**^	0.12^**^	0.63^**^					
5. Life satisfaction T3	4.56	0.93	0.12^**^	0.03	0.59^**^	0.70^**^				
6. Gratitude T1	5.91	0.94	0.05^*^	0.04	0.53^**^	0.46^**^	0.41^**^			
7. Gratitude T2	5.92	0.91	0.05	0.04	0.45^**^	0.55^**^	0.45^**^	0.71^**^		
8. Gratitude T3	5.95	0.87	0.03	0.01	0.38^**^	0.49^**^	0.54^**^	0.67^**^	0.74^**^	

*T1, Time 1; T2, Time 2; T3, Time 3*.

* *p < 0.05*;

** *p < 0.01*.

#### Measurement Model and Invariance Test

First, we tested a six-factor measurement model where we constrained all the gratitude factor loadings as well as all the life satisfaction factor loadings to be equal across the three waves. As suggested by Jöreskog (1979), we incorporated auto-correlated error terms for the observed indicators, and we allowed all latent variables to co-vary freely. The model fit was acceptable: *χ*^2^(465) = 1020.632, *p* < 0.001, CFI = 0.937, RMSEA = 0.041. Then, we tested a baseline model where no constraints were imposed. The model fit was also acceptable: *χ*^2^(447) = 993.733, *p* < 0.001; CFI = 0.938; RMSEA = 0.041. Finally, we compared both models. According to Cheung and Rensvold (2002), the assumption of invariance is tenable if the reduction in CFI, when constraints are imposed, is less than 0.01. Here, the change in CFI met this criterion (ΔCFI = 0.001). Despite Cheung and Rensvold (2002) is a widely accepted criterion, recent literature (e.g., Koomen et al., 2012) has highlighted the importance of relying on multiple criteria for testing invariance. The assumption of invariance is also supported when the difference in RMSEA is lower than 0.01 (Chen, 2007) and the constrained model has an expected cross-validation index (ECVI) smaller than the unconstrained model (Browne and Du Toit, 1992; Ruiz et al., 2017). In our case, the change in RMSEA (ΔRMSEA = 0.00) and the change in ECVI (ΔECVI = −0.05) met both criteria. Therefore, it can be concluded that the pattern of factor loadings was invariant across waves for both gratitude and life satisfaction. Hence, we maintained these constraints in all structural models reported below.

#### Confirmatory Factor Analysis Analyses

The definition of gratitude as a life orientation opens the possibility that both gratitude and life satisfaction belong to one single factor. Thus, we performed a confirmatory factor analysis (CFA) in order to examine the factorial validity of the measures in each assessment time. At T1, results showed that the collapsed model [11 indicators; *χ*^2^(44) = 881.45, *p* < 0.001] is significantly worse than a model where gratitude (six indicators) and life satisfaction (five indicators) were modeled as two different latent variables [*χ*^2^(43) = 271.26, *p* < 0.001], Δ*χ*^2^(1) = 610.19, *p* < 0.001. At T2, the collapsed model [*χ*^2^(44) = 356.74, < 0.001] is significantly worse than the two-factor model [*χ*^2^(43) = 111.78, *p* < 0.001], Δ*χ*^2^(1) = 244.96, *p* < 0.001. At T3, the collapsed model [*χ*^2^(44) = 399.07, *p* < 0.001] is significantly worse than the two-factor model [*χ*^2^(43) = 185.38, *p* < 0.001], Δ*χ*^2^(1) = 213.70, *p* < 0.001. Our results show that gratitude and life satisfaction are two different constructs, replicating the findings of McCullough et al. (2002).

#### Longitudinal Analysis

**Figure 1:**
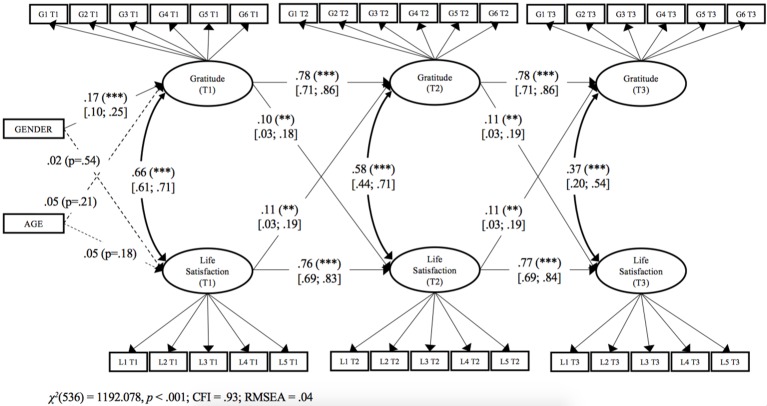
Study 1. Structural longitudinal model for the associations between gratitude and life satisfaction. Coefficients shown are standardized paths. Error terms and loadings are not shown to enhance visual clarity. Loading are all between 0.40 and 0.9 (*p* < 0.001). T1: Time 1; T2: Time 2; and T3: Time 3. Gi, Gratitude item i. Li, Life satisfaction item i. Solid lines = significant paths. Dashed line = not significant paths. Confidence intervals are reported in square brackets for significant paths. ^***^p < 0.001; ^**^p < 0.01.

We tested a structural cross-lagged reciprocal model to determine the relationships between gratitude and life satisfaction over time. Following Ribeiro et al. (2011), we controlled this by gender and age. We allowed the two latent variables (life satisfaction and gratitude) to co-vary within each time point, and we modeled lagged paths from each measure to the other two measures at the successive time points. Thus, all constructs were represented as potential antecedents and as potential consequences of the other constructs, while controlling for stability effects. The model fit was acceptable, *χ*^2^(514) = 1158.80, *p* < 0.001, CFI = 0.93, RMSEA = 0.04. We constrained all factor loading (measurement invariance) and paths (to maximize statistical power) to be equal between waves, following Unanue et al. (2016). The model fit remained acceptable: *χ*^2^(536) = 1192.08, *p* < 0.001, CFI = 0.93, RMSEA = 0.04, and this more parsimonious model showed no significant loss of fit compared to a model where all factor loadings and structural paths were estimated freely: Δ*χ*^2^(4) = 5.50, *p* = 0.239. Values of *R*^2^ ranged from 0.68 to 0.74 (all *p* < 0.001). Supporting H1, we found that gratitude at T1 was a positive prospective predictor of life satisfaction at T2: *β* = 0.10 (95% CI: 0.03, 0.18), *p* < 0.01. Supporting H2, life satisfaction at T1 was a positive prospective predictor of gratitude at T2: *β* = 0.11 (95% CI 0.03, 0.19), *p* < 0.01. We also found that life satisfaction at T1 was a positive prospective predictor of life satisfaction at T2 [*β* = 0.76 (95% CI 0.69, 0.83), *p* < 0.001] and gratitude at T1 was a positive prospective predictor of gratitude at T2 [*β* = 0.78 (95% CI 0.71, 0.86), *p* < 0.001]. Gender was positively related to gratitude: *β* = 0.17 (95% CI 0.10, 0.25), *p* < 0.001. No other significant paths were found. Details may be found in Figure 1^4^. Finally, we constrained the path from gratitude to life satisfaction as well as the path from life satisfaction to gratitude to be equals. The model fit remained acceptable: *χ*^2^(537) = 1192.17, *p* < 0.001, CFI = 0.93, RMSEA = 0.04, and it did not show significant differences in comparison with the previous model, Δ*χ*^2^(1) = 0.093, *p* = 0.760. Thus, the strength of the link from gratitude to life satisfaction is not significantly different from the strength of the link from life satisfaction to gratitude.

## Study 2

Voelkle et al. (2012) show that over shorter periods of time, different lags (e.g., 1 versus 2 months) may yield different conclusions about the strength of the effect sizes. Thus, in order to establish robustness, Study 2 tested the same hypotheses as Study 1 but used a larger sample size as well as a longer period of time between waves (3 months).

### Method

#### Participants and Procedure

Study 2 was conducted in accordance with the same ethical standard and followed the same procedure as Study 1. A three-wave cross-lagged longitudinal design with 3 months between each wave was employed.

In total, 1,841 Chilean working adults (54.9% male) between the ages of 21 and 71 years (mean age = 36.94; SD = 8.59) completed T1 measures. At T2, 979 participants (56.0% male) between the ages of 23 and 75 years (mean age = 38.57; SD = 9.56) answered T2 measures (53.2% of Wave 1). At T3, 700 participants (54.0% male) between the ages of 24 and 72 (mean age = 38.96; SD = 9.77) completed T3 measures (38.0% of Wave 1). Finally, 421 respondents (54.4% male) between the ages of 24 to 71 (mean age = 38.70; SD = 9.63) answered the three waves (22.9% of Wave 1). Those who completed only T1 (*N* = 1,420) did not differ significantly in gender {[*χ*^2^(2)] = 0.64, *p* = 0.730} from those who participated in the three waves (*N* = 421). However, participants differed in age [t(609.84) = −4.47, *p* < 0.001], gratitude [t(762.96) = −2.14, *p* = 0.033] and life satisfaction [t(740.29) = −2.41, *p* = 0.016]. Our analysis suggests that younger participants as well as respondents with lower gratitude and life satisfaction were especially likely to drop the survey. Little’s MCAR test (Little, 1988) showed that missing data were not completely at random {[*χ*^2^(98)] = 150.512, *p* < 0.001}. Thus, following the recommendations of Newman (2014), we used FIML to deal with missing data.

The sensitivity power test indicated that our study was sufficiently powered to detect a predictor with a population effect size of *f^2^* = 0.018, representing a small effect (Cohen, 1992). The distributions were adequate for all constructs (George and Mallery, 2010). Skew values were appropriate for gratitude (T1: −1.01; T2: −0.87; and T3: −0.85) and life satisfaction (T1: −0.67; T2: −0.71; and T3: −0.53). Kurtosis values were also appropriate for gratitude (T1: 1.21; T2: 0.46; and T3: 0.64) and life satisfaction (T1: 0.21; T2: 0.45; and T3: 0.07).

#### Measures

We used the same measures as in Study 1. Cronbach’s alphas were good for gratitude at T1 (0.78), T2 (0.77), and T3 (0.77) as well as for life satisfaction at T1 (0.88), T2 (0.89), and T3 (0.88).

### Results

We followed the same procedure as in Study 1 for testing our hypotheses. Descriptive statistics and inter-correlations for all Study 2 variables are shown in Table 1. Again, we used SEM and latent variables to reduce the biasing effects of measurement error (Finkel, 1995).

#### Measurement Model and Invariance Test

We followed the same procedure as in Study 1. First, we tested a six-factor measurement model where we constrained all the gratitude factor loadings as well as all the life satisfaction factor loadings to be equal across the three waves. We incorporated auto-correlated error terms for the observed indicators (Jöreskog, 1979) and allowed all latent variables to co-vary freely. The model fit was acceptable, *χ*^2^(465) = 1376.928, *p* < 0.001, CFI = 0.954, RMSEA = 0.033. Then, we tested a baseline model where no constraints were imposed. The model fit was also acceptable: *χ*^2^(447) = 1335.096, *p* < 0.001; CFI = 0.955; RMSEA = 0.033. Finally, we compared both models. Because the change in CFI was less than 0.01 (ΔCFI = 0.001), the difference in RMSEA was lower than 0.01(ΔRMSEA = 0.00), and the constrained model had an ECVI smaller than the unconstrained model (ΔECVI = −0.02); therefore, it can be concluded that the patterns of factor loadings were invariant across waves for both gratitude and life satisfaction (Browne and Du Toit, 1992; Cheung and Rensvold, 2002; Chen, 2007; Ruiz et al., 2017). Hence, we maintained these constraints in all structural models reported below.

#### Confirmatory Factor Analysis

CFA showed, again, that gratitude and life satisfaction are different constructs. At T1, the collapsed model [11 indicators: *χ*^2^(44) = 1967.79, *p* < 0.001] was significantly worse than a model where gratitude (six indicators) and life satisfaction (five indicators) were modeled as two different latent variables [*χ*^2^(43) = 506.27, *p* < 0.001], Δ*χ*^2^(1) = 1461.52, *p* < 0.001. At T2, the collapsed model [*χ*^2^(44) = 1151.50, *p* < 0.001] was significantly worse than the two-factor model [*χ*^2^(43) =303.02, *p* < 0.001], Δ*χ*^2^(1) = 848.48, *p* < 0.001. At T3, the collapsed model [*χ*^2^(44) = 628.13, *p* < 0.001] was significantly worse than the two factor model [*χ*^2^(43) =194.77, *p* < 0.001], Δ*χ*^2^(1) = 433.36, *p* < 0.001.

#### Longitudinal Analysis

**Figure 2:**
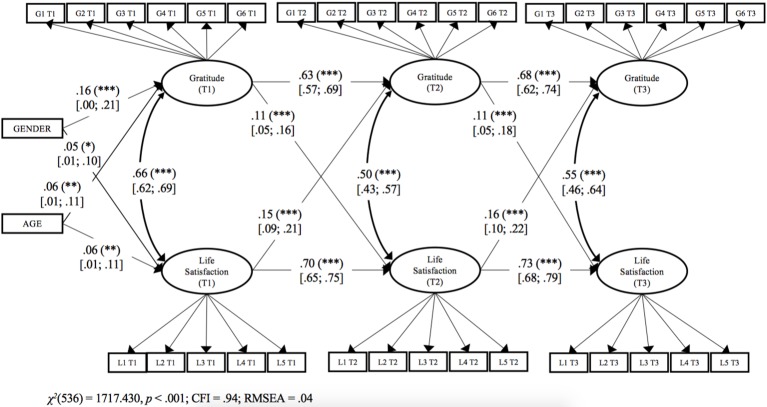
Study 2. Structural longitudinal model for the association between gratitude and life satisfaction. Coefficients shown are standardized paths. Error terms and loadings are not shown to enhance visual clarity. Loading are all between 0.40 and 0.9 (*p* < 0.001) T1: Time 1, T2: Time 2, and T3: Time 3. Gi, Gratitude item i; Li, Life satisfaction item i. Solid lines = significant paths. The confidence intervals are reported in square brackets for significant paths. ^***^p < 0.001; ^**^p < 0.01; *p < 0.05.

We replicated the same cross-lagged model we tested in Study 1. The model fit for our final model (loadings and paths constrained to be equal across waves) was acceptable, *χ*^2^(536) = 1717.43, *p* < 0.001, CFI = 0.94, RMSEA = 0.04 and showed no significant loss of fit compared to a model where all structural paths were estimated freely, Δ*χ*^2^(4) = 6.06, *p* = 0.194. The values of *R*^2^ ranged from 0.54 to 0.67 (all *p* < 0.001). The significant paths from this model are shown in Figure 2. Supporting H1, we found that gratitude at T1 was a significant and positive prospective predictor of life satisfaction at T2, *β* = 0.11 (95% CI 0.05, 0.16), *p* < 0.001. Supporting H2, life satisfaction at T1 was a significant and positive prospective predictor of gratitude at T2, *β* = 0.15 (95% CI 0.09, 0.21), *p* < 0.001. We also found that life satisfaction at T1 was a positive prospective predictor of life satisfaction at T2 [*β* = 0.70 (95% CI 0.65, 0.75), p < 0.001] and gratitude at T1 was a positive prospective predictor of gratitude at T2 [*β* = 0.63 (95% CI 0.57, 0.69), *p* < 0.001]. Gender was significantly and positively related to gratitude, *β* = 0.16 (95% CI 0.00, 0.21), *p* < 0.001 and to life satisfaction, *β* = 0.05 (95% CI 0.01, 0.10), *p* < 0.05, while age was significantly and positively related to gratitude, *β* = 0.06 (95% CI 0.01, 0.11), *p* < 0.01 and to life satisfaction, *β* = 0.06 (95% CI 0.01, 0.11), *p* < 0.01. No other significant path was found. Finally, we constrained the paths from gratitude to life satisfaction, and the paths from life satisfaction to gratitude to be equal. The model fit remained acceptable, *χ*^2^(537) = 1717.92, *p* < 0.001, CFI = 0.94, RMSEA = 0.04, and it did not show significant differences in comparison with the previous model, Δ*χ*^2^(1) = 0.093, *p* = 0.760. Thus, the strength of the link from gratitude to life satisfaction is not significantly different than that from life satisfaction to gratitude.

## Discussion

Research has extensively shown that gratitude and life satisfaction are associated with several indicators of a better life (Wood et al., 2010; Diener and Tay, 2017), but surprisingly, it has remained unknown until now how both constructs relate to each other over time. In addition, despite strong evidence for the gratitude—SWB link in the Western world, cross-cultural research has questioned the results in non-Western countries (Boehm et al., 2011; Layous et al., 2013; Shin et al., in press). In addition, most research into the mentioned link has focused mainly on students and young populations. Based on previous research gaps, we conducted two longitudinal studies, aiming to complement previous experimental and cross-sectional evidence in order to clarify the origin of the link between gratitude and life satisfaction. We tested a reciprocal model, among two large samples of Chilean working adults, using three-wave cross-lagged panel designs with 1 month (Study 1) and 3 months (Study 2) between waves. In both studies, we found that a person with higher than average gratitude at T1 is likely to show higher than average life satisfaction at T2, controlling the stability effect of life satisfaction at T1. In addition, a person with higher than average life satisfaction at T1 is likely to show higher than average gratitude at T2, controlling the stability effect of gratitude at T1. Our data also show that the effect of gratitude on life satisfaction is as strong as—and equally important for the dynamic of human wellness—as the effect of life satisfaction on gratitude. We found these results even when controlling age and gender.

Our findings complement previous experimental and cross-sectional studies, thus providing critical evidence about the benefits of both gratitude and life satisfaction for improving people’s quality of life. Gratitude may help to increase life satisfaction, which is a key element of people’s wellness and functioning. However, the power of life satisfaction also goes beyond what is already known (Diener et al., 2017; Diener and Tay, 2017) as life satisfaction also predicts gratitude. This is the most novel aspect of our paper, as by linking life satisfaction to gratitude over time, our results open the possibility for enriching life satisfaction conceptualization. Besides being understood as cognitive evaluation, life satisfaction would be an experience in itself, full of thankfulness, emotions, and positive ways of living our lives.

Previous literature has highlighted the role of culture in the link between gratitude and SWB (Boehm et al., 2011; Shin et al., in press). However, previous cross-cultural research has only contrasted Western American and Eastern Asian populations, which is not enough to reflect the variety of cultures around the world (Vignoles et al., 2016). Further, we assessed a sample of Chileans, from a Latin American country. Chile presents important differences with Western and Eastern countries previously studied (e.g., economic development, religious heritage, and individualism), adding more diversity to gratitude research across the world. Our results support the bi-directional link between gratitude and life satisfaction in this unexplored non-Western, Latin American context.

Interesting findings emerge when inspecting the longitudinal effects of age and gender in our outcome variables. Both studies showed, consistently, that gratitude is significantly higher for women than men, whereas Study 2 also found that women are more likely to experience life satisfaction. Moreover, Study 2 also showed that older participants report higher levels of both life satisfaction and gratitude. Further research may explore the psychological process behind these results, which may in turn help policy makers and clinicians to design better interventions to improve people’s lives at particular stages.

Our findings yield practical implications, e.g., for organizations, as our participants are all working adults. Companies may start a reciprocal process of happiness and flourishing by creating the necessary conditions for fostering either employees’ gratitude or life satisfaction. Previous research has found a significant association between job satisfaction and life satisfaction (Unanue et al., 2017). Thus, by improving working conditions, leaders may increase worker satisfaction, and thus, life satisfaction. This process may naturally lead employees to feel more grateful, thus reinforcing life satisfaction and allowing an upward spiral in human wellness.

Despite the positive loop, it is important to notice that a lower level of gratitude may also lead to a negative spiral in human wellness through the reinforcing effect of lower life satisfaction. For example, if companies affect people’s lives and/or job satisfaction negatively, they may start a negative process in those individuals’ well-being through a lack of gratitude. Indeed, the virtuous circle between gratitude and life satisfaction could become a vicious one. This highlights how important it is to develop strategies for improving gratitude and life satisfaction over time. Otherwise, people’s mental health and well-being could be at risk.

In sum, our results show that gratitude and life satisfaction are both prospectively and positively related to each other over time. Higher levels of gratitude may lead to an increase in life satisfaction, which in turn may increase gratitude, thus enabling a spiral of human flourishing. To the best of our knowledge, this is the first research that has shown these patterns of results, thereby allowing a better interpretation of previous cross-sectional and experimental findings.

### Limitations

Some limitations in this research should be acknowledged. First, our measures were all self-reported and shared method variance could potentially have inflated the correlations between gratitude and life satisfaction within each wave. However, self-reports of one’s experience are the most valid way of measuring gratitude and life satisfaction, since these are facets of people’s subjective experience. In addition, we took several *a priori* precautions to mitigate a common method bias. For example, we adapted highly validated measures for our constructs. Moreover, shared method variance within each measure was reduced within the stability paths that we controlled while testing the lagged paths that formed the main focus of our research. Finally, we protected respondent anonymity and informed participants that there were no right or wrong answers (Podsakoff et al., 2003; Conway and Lance, 2010). Nonetheless, despite these previous precautions, future studies might supplement the current findings with alternative methods, such as implicit measures of gratitude and life satisfaction, as well as proximal mechanisms such as biomarkers, as suggested by Davis et al. (2016).

Second, by providing evidence of temporal precedence, the prospective bi-directional longitudinal link between gratitude and life satisfaction reported in our research substantially strengthens the hypothesized causal relationships between both constructs. However, these results do not provide conclusive evidence for causality. A third variable may be involved. Thus, future research should investigate the role of possible mediators (such as the ones we explicitly theorized across the paper) in the link we studied. Third, we found small lagged paths between gratitude and life satisfaction. However, effect sizes in CLPMs are typically small because most of the variance is captured by the stability paths. Fourth, although we sampled adults from a non-Western country, the participants were all from Chile. Thus, we should be careful about generalizing these results to different non-Western cultures and populations.

Fifth, it would be important to attempt to reduce attrition rates in future research. However, as the review by Wood et al. (2010) has shown, attrition in online studies of gratitude is “commonly very high” (p. 8). Indeed, “the law of attrition” is almost a fact in all data collection without human contact, and “high dropout rates may be a natural and typical feature” (Eysenbach, 2005, p. 1). Sixth, CLPMs are not exempt from criticism. For example, one potential limitation is that they do not explore how variables are evolving and changing over time, which may be useful for understanding individual differences. However, this issue is beyond our aim here. We were only interested in prospective directions. Further research should also explore our hypothesis using, for example, latent growth models aiming to test within-person changes.

Seven, we recognized the possibility that our studies may suffer from uncareful responses, which may affect the quality of the data collected (Chandler et al., 2014). However, the main constructs used in the present paper showed adequate reliabilities and were invariant across time, allowing us to think that most people provided true and careful answers. Nonetheless, future research should follow Porter et al. (2019, p. 19) suggestions, in terms of “create unique attention checks” and “use conventional attention checks to identify and potentially remove responses provided by careless”. Eighth, the quantitative nature of this study could limit the potential understanding and the complexity of the phenomena we explored. Further, qualitative methodology may help to complement our findings, helping to understand the underlying process between gratitude and life satisfaction in more detail.

Nine, we advocated for several underlying mechanisms that may explain the virtuous circle between gratitude and life satisfaction. First, e.g., drawing on BBT (Fredrickson, 2013), gratitude may enhance a positive affectivity (Watkins et al., 2003) that would foster congruent positive cognitions which, in turn, would improve positive evaluations that people make about their lives (Watkins, 2004), thereby enabling a positive spiral in human functioning. Second, the positive spiral between gratitude and life satisfaction might also be explained due to the emotional benefits that individuals experience when something is interpreted as a gift (McCullough et al., 2001; Watkins, 2004). Indeed, positive cognitions and positive affects linked to life satisfaction and gratitude respectively, could gradually generate a cognitive bias that would impact on the availability of people’s memories, thoughts, feelings, and perceptions of life events. In line with this, Watkins (2004) suggests that gratitude could promote a mood-congruent memory bias that could enhance both the encoding and retrievability of positive experiences, increasing the elaboration of positive information. Lambert et al. (2012) support previous theorization, proposing that individuals high in trait gratitude are more likely to reframe negative or neutral events in a positive way which, in turn, lead them to experience fewer depressive symptoms. Third, Watkins (2004) argues that happy people are “more likely to acknowledge the good intentions of a giver” (p. 184). In other words, people with high SWB would be more prompt to attribute positive intentions from others and, in doing so, to experience gratitude. Thus, the more someone values the gift, or the more people recognize the benevolence acts of a giver, the more likely he/she will feel grateful (Tesser et al., 1968; Watkins, 2004). We suggest that these positive attributions could also have a positive effect on the quality of social contacts, which could be strengthened due to the consequent gratitude of the beneficiary and his or her motivation to act in a reciprocal way towards the giver (i.e., helping him or her). This is consistent with previous findings that identify social support as a mediator between gratitude and life satisfaction (Wood et al., 2010; Kong et al., 2015) as well as between SWB and the quality of the individual’s friendship (Diener et al., 1999). Fourth, different aspects of gratitude may act as a catalyst from one to another. Further, cognitive aspects of gratitude such as mood-congruent elaboration and cognition (Watkins, 2004) could be followed by noticing and appreciating the positive in the world, which in turn, may be validated by social comparison and experiences (e.g., perception, attribution, and experiences) reinforcing positive mood-congruent cognitions. However, despite previous mechanisms possibly playing a key role in the reciprocal link between gratitude and life satisfaction, we did not test them. Therefore, future research may expand on these underlying psychological processes.

Then, and finally, we acknowledge that in this paper we only investigated the link between gratitude and the “bright” side of human experiences (i.e., life satisfaction). However, we strongly encourage future research to explore the link between gratitude and the “dark” side of people’s mental health (i.e., depression). Based on our findings, we would expect a negative reciprocal link between gratitude and depression. However, to the best of our knowledge, only Wood et al. (2008) have examined this reciprocal relationship. In two studies, the authors found a significant and negative link from gratitude to depression, but the reverse hypothesis was not supported. Methodological issues may help to understand these unexpected results. We think that there is a chance that the small sample sizes in both studies (156 and 87 participants, respectively) were not powerful enough for the sophisticated and complex SEM longitudinal models Wood et al. (2008) tested. This issue may play a role in the non-significant findings from depression to gratitude. In addition, only young participants going through the same life transition were assessed, which limits the variability in the data collected as well as the generalization of the results. We encourage the replication of findings of Wood et al. (2008). Patients diagnosed with clinical depression tend to focus more on negative than on positive thoughts and have fewer resources to appreciate the positive and good in life. Therefore, we expect that by using larger sample sizes, adult populations, and ideally, different cultures, yield results which show that higher (lower) levels of depression may lead to lower (higher) levels of trait gratitude.

## Conclusion

Violeta Parra wrote one of the most famous Chilean songs almost 50 years ago: Thanks to life. Her gratitude used to come from her life satisfaction, nut research has neglected the possibility of this link. Could this be possible? To date, research has only claimed a link from gratitude to life satisfaction, not the reverse. Notably, we found that gratitude and life satisfaction are mutually linked to each other in a “circle of virtue”. Violeta was right!

## Data Availability Statement

The datasets generated for this study are available on request to the corresponding author.

## Ethics Statement

The studies involving human participants were reviewed and approved by Comité de Ética universidad Adolfo Ibañez. The patients/participants provided their written informed consent to participate in this study.

## Author Contributions

All authors listed have made a substantial, direct, and intellectual contribution to the work. The original idea, as well as the data collection was developed by WU. All authors wrote several sections of the out initial analysis draft, carried and interpreted results. All authors wrote, read, and revised the final paper and approved it for publication collaboratively.

### Conflict of Interest

The authors declare that the research was conducted in the absence of any commercial or financial relationships that could be construed as a potential conflict of interest.

## Footnotes

^1^Study 1 and Study 2 are part of a large project on happiness and well-being, funded by the Chilean Government and KU Leuven. We collected several other measures regarding life and work, but they are not relevant to the present research.

^2^We use full information maximum likelihood (FIML), because this procedure outperforms traditional techniques regarding parameter estimation bias, model fit and parameter estimation efficiency (Peters and Enders, 2002). In addition, FIML shows unbiased and more efficient estimates compared with other methods of imputation such as listwise deletion, pairwise deletion and similar response patterns imputation (Enders and Bandalos, 2001). Moreover, FIML generate a lower proportion of convergence failures (Enders and Bandalos, 2001).

^3^Nowadays, there are validated Spanish versions for both gratitude (Langer et al., 2016) and life satisfaction (Bagherzadeh et al., 2018) scales. However, they were not available at the time when we started the data collection. Nonetheless, we have checked them against our back translation, and we do not find any particular difference between the items.

^4^In Study 1 and Study 2, we reported standardized paths only between T1 and T2. Paths between T2 and T3 may be found in their respective figures, but they are similar in significance and magnitude.
